# Sheathless inertial cell focusing and sorting with serial reverse wavy channel structures

**DOI:** 10.1038/s41378-018-0005-6

**Published:** 2018-05-07

**Authors:** Yinning Zhou, Zhichao Ma, Ye Ai

**Affiliations:** Pillar of Engineering Product Development, Singapore University of Technology and Design, Singapore, 487372 Singapore

## Abstract

Inertial microfluidics utilizing passive hydrodynamic forces has been attracting significant attention in the field of precise microscale manipulation owing to its low cost, simplicity and high throughput. In this paper, we present a novel channel design with a series of reverse wavy channel structures for sheathless inertial particle focusing and cell sorting. A single wavy channel unit consists of four semicircular segments, which produce periodically reversed Dean secondary flow along the cross-section of the channel. The balance between the inertial lift force and the Dean drag force results in deterministic equilibrium focusing positions, which also depends on the size of the flow-through particles and cells. Six sizes of fluorescent microspheres (15, 10, 7, 5, 3 and 1 μm) were used to study the size-dependent inertial focusing behavior. Our novel design with sharp-turning subunits could effectively focus particles as small as 3 μm, the average size of platelets, enabling the sorting of cancer cells from whole blood without the use of sheath flows. Utilizing an optimized channel design, we demonstrated the size-based sorting of MCF-7 breast cancer cells spiked in diluted whole blood samples without using sheath flows. A single sorting process was able to recover 89.72% of MCF-7 cells from the original mixture and enrich MCF-7 cells from an original purity of 5.3% to 68.9% with excellent cell viability.

## Cell sorting: Effective separation in wavy channels

A technique for sorting tiny objects based on their size as they flow through narrow channels offers a simple system that could be used to separate cells and smaller bodies such as blood platelets. Separating different cells and other components of biological fluids is a vital aspect of medical research toward the development of new therapies. All existing methods have limitations, and improved techniques are eagerly sought. Ye Ai and colleagues at Singapore University of Technology and Design developed and tested a simple method based on the forces particles experience as they flow through channels with semi-circular sections linked in repeatedly reversing directions. The researchers demonstrated the general performance of their technique using fluorescent microspheres. They then successfully separated cancer cells from blood. The method could allow fast and efficient cell-sorting in many biomedical applications.

## Introduction

Precise manipulation and separation of cells at the microscale are an essential technology for enabling biological studies and exhibit immense commercial potential in the bioengineering and pharmaceutical industries. In the past two decades, various microfluidic cell sorting technologies have been developed and can be classified as active and passive methods. Conventional active methods generally apply external acoustic^1–5^, electric^6–10^ and magnetic^11–13^ fields, taking advantage of the powerful ability of highly accurate cell separation. However, the extensive utilization of active cell separation methods in practical applications is hampered owing to complicated device fabrication and integration and relatively low throughput, especially when the processing of a large sample volume, i.e., on the order of a few milliliter, is required to isolate extremely low-abundance biological particles. Passive cell sorting techniques mainly include size-based microfiltration^14,15^, deterministic lateral displacement (DLD)^16–18^ and inertial focusing. As far back as 1961, Segré and Silberberg^19^ first observed that particles would spontaneously form an annulus pattern along a cylindrical pipe in a laminar flow regime (tubular pinch effect), which arises from the balance between two opposing inertial lift forces. This lateral migration to deterministic equilibrium positions is known as the inertial focusing phenomenon. Inertial focusing has emerged as one of the most powerful and precise cell manipulation techniques in microfluidics since 2007^20^ and has then gradually begun to attract great attention in the microfluidics research community because of its high throughput, low-energy consumption, simple device structure and friendly fabrication procedures^21–23^.

Inertial focusing is a passive microfluidic manipulation technology in which the size-selective manipulation highly depends on the channel geometry. Various channel geometries have been adopted to demonstrate inertial focusing, including straight^24–27^, curved/serpentine^28–31^, asymmetric curves^29,32,33^, spiral^27,34–36^ and contraction/expansion^37–40^, each of which exhibits different inertial focusing behavior^21^. Microfluidic channels with curvilinear or expansion-constriction features can produce a Dean secondary flow perpendicular to the main flow direction. The generation of the Dean flow results from the inertia mismatch of continuous flow in the center and near-wall regions, which is typically counter-rotating Dean vortices along the cross-section of the channel. The Dean secondary flow accordingly produces a Dean drag force that can be used to balance the inertial lift force and thus provides flexibility to control a particle’s equilibrium positions^41^. In particular, the Dean drag force and inertial lift force scale with the particle size very distinctively, which leads to distinct equilibrium positions of differently sized particles for particle sorting in continuous flows^42^. The secondary Dean flow also helps reduce the number of equilibrium positions, making sample collection more convenient.

As a pluripotent microfluidic manipulation method, inertial focusing has been applied in multiple applications, such as sheathless alignment in flow cytometry^30,43^, size-dependent cell separation^36,44,45^, deformability-dependent cell separation^46^, rare cell separation^32,34,40,47^, bacteria isolation^26^, platelet separation^29^, plasma extraction^48^ and solution exchange^40,49^, among others. Notably, circulating tumor cells (CTCs) are malignant cancer cells shed from a primary tumor (or a tumor after metastasis) that undergo an epithelial–mesenchymal transition (EMT) and then intrude into the circulatory system. CTCs are considered a prerequisite of tumor metastasis, and the ability to capture and analyze CTCs enables the early diagnosis of cancer and systematic study of cancer metastasis. However, CTCs are extremely rare in the bloodstream (i.e., tens of CTCs in 1 ml whole blood sample^50^); therefore, to meet the demands of practical research and clinical use, CTC sorting technologies need to fulfill the requirements of high throughput, purity and capture rate. Since inertial focusing has the ability to process samples in a high-throughput manner, there has been an increasing interest in developing high-throughput inertial sorting or enrichment technology. For example, the spiral channel is a design that has been extensively studied and applied to inertial cell focusing and sorting, for example, rare cell isolation^35,51,52^ (e.g., CTCs), specific cell type separation^27,36,44^ and single cell encapsulation^53^. Warkiani et al. achieved at least an 85% recovery rate of cancer cells spiked in lysed blood sample using a spiral inertial device combined with sheath flow^54^ and >80% recovery of cancer cells spiked in whole blood through a slanted spiral channel with sheath flow^51^. Sun et al. obtained an 88.5% recovery rate of cancer cells spiked into whole blood with a double spiral microchannel^35^.

In this work, we present a novel geometric channel design, an asymmetric reverse wavy microchannel, for sheathless inertial particle focusing and cell sorting. The goal of using sheathless microfluidic devices is to minimize the use of fluid pumps, which inevitably cause increased power and buffer solution consumption, as well as to avoid the complicated procedure of sheath flow control. The advantages of sheathless microfluidics have been well demonstrated in previous studies involving the processing of viscoelastic solutions by Liu et al.^55,56^ and Li et al.^57^. Although multiple cross-section shapes, such as trapezoids^44^, circles, semicircles and triangles^58^, have been studied, we chose a classic rectangular cross-section design because of its simple fabrication process. The inertial focusing behavior of six fluorescent micron-sized particles (15, 10, 7, 5, 3 and 1 μm) in three channel pattern designs was experimentally examined. It was found that the minimum particle size for effective inertial focusing is between 1 and 3 μm. On the basis of these experimental studies, we have identified an optimized channel design to fulfill our requirements of separating cancer cells from whole blood samples. To demonstrate the application potential of this novel device design, diluted whole blood samples spiked with breast cancer cells to mimic clinical CTC samples were used to test the sorting performance of our inertial microfluidic device. A single sorting process is able to recover ≥89% cancer cells and increase the purity of cancer cells by 13 times. Compared with previous inertial sorting devices, our novel design with extremely sharp-turning subunits can effectively focus cells as small as 3 μm and can thus effectively separate the three major blood cell types (i.e., red blood cells, white blood cells, and platelets) from cancer cells without the use of sheath flows. In addition, the repeated wavy units are arrayed in a linear direction, which enables easier horizontal (2D) and vertical (3D) parallelization of multiple channels for handling large-volume samples.

## Concept and operating principle

When a solid particle is flowing along a bounded straight channel in an intermediate Reynolds number regime (~100 > *Re* > ~1), in addition to the viscous drag force exerted on the particle along the main flow direction, there are four types of inertial lift forces acting on the particle perpendicular to the main flow^22^: (i) the Magnus force due to particle slip-rotation; (ii) the Saffman force due to particle slip-shear; (iii) the shear gradient-induced lift force due to the curvature of the fluid velocity profile (pointing from the particle to the wall), and (iv) the wall-induced lift force due to interaction between the particle and wall (pushing the particle away from the wall). Among these forces, the Magnus force and Saffman force are typically much smaller than the other two lift forces and can usually be ignored in microfluidic sorting applications. The balance between the shear gradient- and wall-induced inertial lift forces results in the tubular pinch effect along a cylindrical pipe observed by Segré and Silberberg.^19^ According to Asmolov’s model^42,59^, the net inertial lift force consisting of the two major lift forces can be expressed as follows,

1 $${\mathrm{near}}\,{\mathrm{the}}\,{\mathrm{center:}}\,F_L = \frac{{f_L\rho _fU^2a^4}}{{H^2}}$$

2 $${\mathrm{near}}\,{\mathrm{the}}\,{\mathrm{wall:}}\,F_L = \frac{{f_L\rho _fU^2a^6}}{{H^4}}$$

In the above equations, *f*_*L*_ refers to the lift coefficient, which is usually taken as 0.5^20^ when the Reynolds number Re<100, and $\rho _f$, *U* and *a* refer to the fluid density, fluid velocity and particle diameter, respectively. *H* here is defined as the hydraulic diameter and is calculated in a rectangular channel as $2wh/(w + h)$, in which *w* refers to the channel width and *h* refers to the channel height of the cross-section.

The steady-state incompressible Navier–Stokes equation (Eq. 3) and continuity equation (Eq. 4) are used to describe the fluid flows inside the microchannel. The term on the left hand side of Eq. 3 is the fluid inertia, which produces the Dean secondary flow in the sharp-turning subunits,

3 $$\rho _f\left( {\vec u \cdot \nabla } \right)\vec u = - \nabla p + \mu \nabla ^2\vec u$$

4 $$\nabla \cdot \vec u = 0$$

To quantify the relationship between the inertial force and viscous force acting on the fluid, the channel Reynolds number, Re_c_, is defined as follows,

5 $$Re_c = \frac{{U_m\rho _f{\mathrm{H}}}}{\mu }$$

where *U*_*m*_ is the maximum flow velocity, *μ* is the flow viscosity, $\vec u$ is the fluid velocity vector and *p* is the fluid pressure.

The introduction of secondary lateral flows, for example, a curvature-induced Dean flow in an intermediate Reynolds number regime, enables more control of the particle’s equilibrium positions^21^. Due to the mismatch in lateral centrifugal force between the continuous flow in the center and near-wall regions and conservation of mass, two counter-rotating Dean vortices could be generated along the cross-section of the curved channel. A dimensionless Dean number, *De*, is used to characterize the Dean flow strength^41,60^,

6 $$De = Re_c\sqrt {\frac{H}{{2R}}}$$

where *R* refers to the radius of curvature. The magnitude of the Dean flow scales with ${\mathrm{U}}_m^2$ as,

7 $$U_D\sim {\mathrm{De}}^2\frac{\mu }{{\rho _fH}}$$

The Dean flow drags the particle that is perpendicular to the main flow direction, and the Dean drag force can be defined as,

8 $$F_D = 3{\mathrm{\pi \mu a}}U_D\sim \frac{{{\mathrm{\rho }}_fU_m^2aH^2}}{R}$$

The Dean drag force has a linear size scaling, which is different from the size scaling of the net inertial lift force. Therefore, the concurrent effect of the Dean drag force and net inertial lift force results in distinct equilibrium positions of differently sized particles, which enables size-based inertial sorting in a continuous flow.

Two empirical parameters have been found in previous studies of certain channel geometries to guide the design of inertial sorting devices. First, *a*/*H*>0.07 is generally recommended to achieve successful inertial focusing^20^. Another empirical parameter, *R*_*f*_, is the ratio of the inertial lift force to the Dean drag force, defined as below^23^,

9 $$R_f = \frac{{2a^2R}}{{H^3}}$$

When $R_f > \sim 0.08$, the equation indicates that the inertial lift force dominates the Dean drag force. On the contrary, the particle motion is dominated by the Dean flow rather than the inertial lift force when $R_f < \sim 0.08$. In addition, a too small value of *R*_*f*_ would generate chaotic particle motion instead of deterministic particle focusing.

**Fig. 1:**
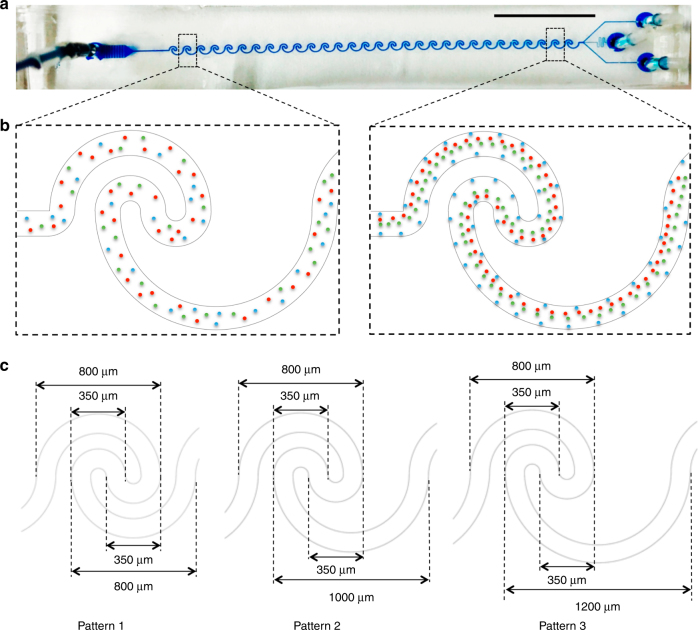
Three different channel designs for inertial focusing with a series of reverse wavy channel units. **a** Photograph of a representative inertial sorting microfluidic device. The injection of the blue dye into the microchannel helps visualize the channel design. Scale bar is 1 cm. **b** Schematic inertial focusing behavior of three differently sized microparticles (3 μm, blue; 10 μm, red; and 15 μm, green) in a single mixed input. **c** Detailed geometric parameters of each pattern. Patterns 1–3 share the same geometric parameters of the upper semicircle (both the outer and inner semicircle) and lower inner semicircle, with the lower outer semicircle increasing by 200 μm. All the channel designs have a width of 125 μm and a height of 40 μm

Figure 1 shows three different channel patterns tested to understand how the radius of the lower outer semicircular affects the inertial particle focusing in these channel geometries. Patterns 1–3 possess an identical upper semicircular design and a different lower outer semicircular size, in which pattern 1 is geometrically symmetric with respect to the center of the unit pattern, while pattern 2 and 3 exhibit certain degrees of geometric asymmetry. All three channel designs use a single inlet design, and the main channel has a width of 125 μm, a height of 40 μm, and a low aspect ratio design (AR=*h*/*w*=0.32). The widths of the three outlet branches for all patterns are 80 μm, 45 μm and 80 μm, respectively. The diameter of the inlet and outlet reservoirs is 1.5 mm. Figure 1a shows a representative microfluidic channel with serial reverse wavy channel structures, in which the randomly distributed particles at the inlet could be deterministically focused into differential tight streaks when exiting the channel based on the particle size (Fig. 1b). Detailed geometric parameters of these pattern designs are shown in Fig. 1c.

## Materials and methods

### Device fabrication

The three different microchannels were fabricated using a standard polydimethylsiloxane (PDMS) soft-lithography process, in which the master molds for PDMS casting were fabricated with SU-8 (SU-8 2025, MicroChem, Newton, MA, USA) on a silicon wafer. The PDMS microchannel layer and an ultrasonically cleaned glass slide were treated with air plasma (Harrick Plasma PDC-32G, Ithaca, NY, USA) to generate hydroxyl functional groups on the surfaces. The treated surfaces were then brought into contact with each other to form a closed microchannel.

### Numerical modeling

A finite element method (FEM)-based numerical simulation was conducted using the laminar flow module with a steady-state study in COMSOL Multiphysics 5.0 (www.comsol.com, USA). The model consists of three reverse wavy channel units with the same geometric dimensions and inlet flow rate as the experiments. The incompressible Navier-Stokes equation (Eq. 3) and continuity equation (Eq. 4) were the governing equations used to simulate the fluid motion inside the microchannel, which could help understand how the Dean secondary flow affects the inertial particle focusing. The boundaries other than the inlet and the outlet were set as a non-slip condition. The inlet velocity at a flow rate of 197.60 μl/min (corresponding to the channel Reynolds number Re_c_=40) was calculated, and the maximum Dean flow velocity was also obtained.

### Cell culture

The MCF-7 breast cancer cell line was purchased from the American Type Culture Collection (ATCC Cat. No. HB-72, USA) and was cultured in Dulbecco’s modified Eagle’s medium (DMEM) (Thermo Fisher Scientific, USA) supplemented with 10% fetal bovine serum (FBS, Thermo Fisher Scientific, USA) to provide growth factors and antibiotics including penicillin and streptomycin (Thermo Fisher Scientific, USA) to prevent the growth of bacteria. The cells were subcultured every 2 to 3 days when the monolayer reached 80–90% confluence and maintained at 37 °C and 5% (v/v) CO_2_ in a humidified incubator. The cells were then trypsinized with 0.25% trypsin-EDTA solution (Thermo Fisher Scientific, USA).

### Sample preparation

Fluorescent polystyrene microspheres (15, 10, 7, 5, 3 and 1 μm) were purchased and used without any further modification (Magsphere, USA). All these fluorescent polystyrene particles were diluted with deionized (DI) water containing 0.6% Pluronic F127 (Sigma-Aldrich, USA) to avoid particle agglomeration and adhesion onto the channel wall. The typical particle concentration used in the following experiments was ~6×10^6^ particles per ml. A mixture of 15 μm, 10 μm and 3 μm particles suspended in DI water (with 0.6% F127) was used to demonstrate size-based particle sorting in continuous flow. Cancer cells (MCF-7) were stained with SYTO 9 fluorescent dye (Thermo Fisher Scientific, USA) and mixed with diluted whole blood (final concentration of ~5×10^7^ cells/ml). This cell mixture was used to demonstrate size-based sorting of MCF-7 cancer cells from blood cells using this wavy inertial focusing device.

### Experimental setup

Each individual experiment was conducted with a new microchannel device to avoid cross-contamination and possible clogging by residual particles or bubbles in the used devices. For each experiment, the prepared aqueous sample was continuously infused into the microchannel at flow rates from 49.41 μl/min to 197.60 μl/min (corresponding to Re_c_ from 10 to 40) using a syringe pump. The trajectories of these fluorescent microparticles were recorded using a CCD camera on an inverted microscope (Olympus, CKX53, Japan) to capture the inertial focusing behavior. The motion of single cells in the trifurcated outlet was captured using a high-speed camera (FASTCAM Mini UX100, PHOTRON, Japan) to visualize the cell separation process. The cell contents in the samples before and after inertial sorting were analyzed by a commercial flow cytometer (Accuri C6, Becton Dickinson, CA, USA) to evaluate the sorting performance.

## Results and discussion

### Simulation of fluid flow in the three channel designs

**Fig. 2:**
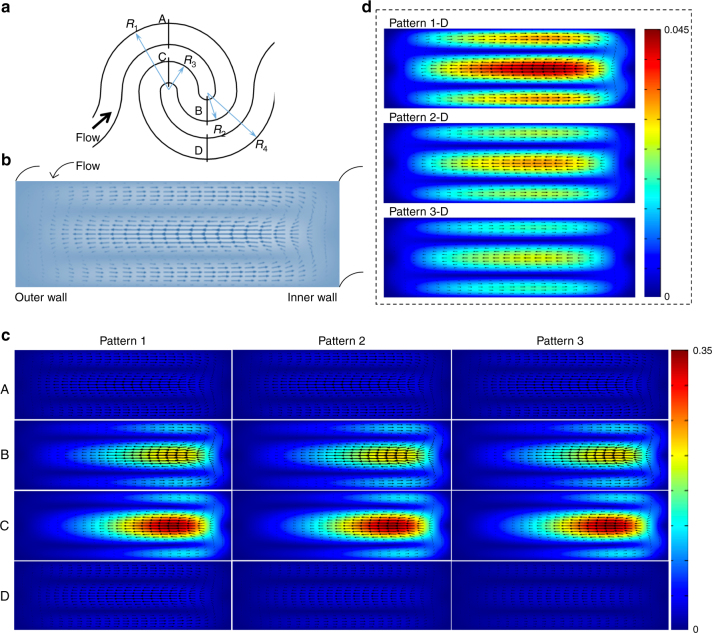
Numerical simulation of the Dean secondary flow at different cross-sections in the three channel designs. **a** Four cross-sections, A-D, selected to visualize the Dean flow along a single wavy channel unit. *R*_1_, *R*_2_, *R*_3_, and *R*_4_ are the radius of curvature of the upper outer semicircle, lower inner semicircle, upper inner semicircle, and lower outer semicircle, respectively. **b** A typical Dean secondary flow (two symmetric counter-rotating vortices) along the channel cross-section. **c** Simulated velocity profile along the four cross-sections, A-D, in the three channel designs. All the left sides denote the outer wall of the channel. The color level represents the magnitude of the Dean flow velocity. **d** Zoomed-in scale of the velocity profile at cross-section D in the three channel patterns

We first simulated the fluid flow in the three different channel designs and were in particular interested in the velocity profile along four cross-sections, A-D, as defined in Fig. 2a. All the simulations were conducted at a flow rate of 197.60 μl/min (Re_c_ = 40). Figure 2b shows a representative flow profile along one of the channel cross-sections, in which the left and right sides are the outer wall (larger radius of curvature) and inner wall (smaller radius of curvature) of the channel, respectively. When the fluid flows through a turning channel, the inertia of the fluid becomes nontrivial at an intermediate channel Reynolds number regime (~100 > Re_c_ > ~1). In the middle region of the channel, the faster moving fluid along the main flow direction tends to move toward the outer wall along the cross-section direction. To conserve the fluid mass in the closed channel, the slower moving fluid near the top and bottom walls tends to move toward the inner wall, generating two symmetric counter-rotating vortices perpendicular to the main flow direction, which is called the Dean secondary flow.

The first column in Fig. 2c shows the Dean secondary flow along the four cross-sections in channel pattern 1, which is designed to be geometrically symmetric with respect to the center of a single wavy channel unit. When the fluid flows through cross-section A, it starts to produce a relatively weak Dean secondary flow along the cross-section. The outer wall in Fig. 2c is always on the left side of the channel cross-section. From cross-section A to B, the fluid flows from the upper outer semicircle to the lower inner semicircle, during which the radius of curvature gradually decreases. As the magnitude of the Dean flow inversely scales with *R*, the Dean secondary flow at cross-section B becomes much more pronounced than that at cross-section A. It is worth mentioning that from A to B, the outer wall is along the same channel side. From cross-section B to C, the fluid flows from the lower inner semicircle to the upper inner semicircle, undergoing the steepest flow turning. Although the two cross-sections have the same radius of curvature, the Dean secondary flow at cross-section C becomes even stronger than that at cross-section B. This result can be intuitively understood by considering that the variations in the radius of curvature from A to B and B to C are different, resulting in a considerable difference in the flow development at B and C. The maximum Dean flow velocities at the four cross-sections are listed in Table 1 for quantitative comparison, which reveals an ~27% relative difference in the Dean flow strength between B and C. In particular, because of this reverse wavy channel design, the outer wall from A to B reverses to the inner wall from B to C along the same channel side, implying that the direction of Dean secondary flow along the cross-section reverses from cross-section B to C. From cross-section C to D, the radius of curvature gradually increases, and the inner wall remains along the same channel side. As a result, the Dean secondary flow becomes weaker when flowing from the upper inner semicircle to the lower outer semicircle. In summary, along a single wavy channel unit, the strength of the Dean secondary flow varies from weak to strong with the strongest Dean vortices in the inner semicircles and then becomes weak again when leaving the single wavy channel unit. In addition, the direction of the Dean secondary flow reverses once through a single wavy channel unit. Although channel pattern 1 is geometrically symmetric with respect to the unit center, the strength of the periodically reversed Dean secondary flow shows a certain degree of asymmetry, particularly along the two inner semicircles.

**Table 1:** Maximum Dean flow velocity at cross-sections A–D in patterns 1, 2 and 3

Maximum velocity (m/s)	A	B	C	D
Pattern 1	0.04231	0.21362	0.29468	0.04216
Pattern 2	0.04286	0.2122	0.28832	0.03219
Pattern 3	0.04165	0.2029	0.27845	0.02599

In contrast to channel pattern 1, patterns 2 and 3 introduce some degree of geometric asymmetry by increasing the radius of curvature of the lower outer semicircle with 100 and 200 μm, respectively. Generally, the Dean flows at cross-sections A, B, C and D show similar velocity profiles. Table 1 quantitatively compares the maximum Dean flow velocity at different cross-sections in the three channel pattern designs. The relative difference in the maximum velocity of the three designs at cross-sections A, B and C is <~5%. As discussed previously, the relative difference in the maximum Dean flow velocity between cross-sections B and C is ~27% in pattern 1. It was found that this relative difference remains ~27% in both pattern 2 and pattern 3, indicating a consistent flow asymmetry from B to C. Since the introduced geometric asymmetry mainly varies the radius of curvature of the lower outer semicircle, it has been found that the relative differences in the maximum velocity at cross-section D between pattern 1 and 2, pattern 1 and 3 are ~23% and ~38%, respectively. To clearly visualize the difference in the Dean flow at cross-section D, we zoom in on the scale of flow velocity, as denoted by the black dashed box shown in Fig. 2d. Clearly, the strength of the Dean vortices at cross-section D decreases from pattern 1 to pattern 3. Because the Dean vortices at cross-section D are approximately seven to ten times weaker than those at cross-section C, the change in the radius of curvature of the lower outer semicircle can be considered a means of fine-tuning the Dean secondary flow in a single wavy channel unit.

### Size-dependent inertial focusing in the three channel designs

**Fig. 3:**
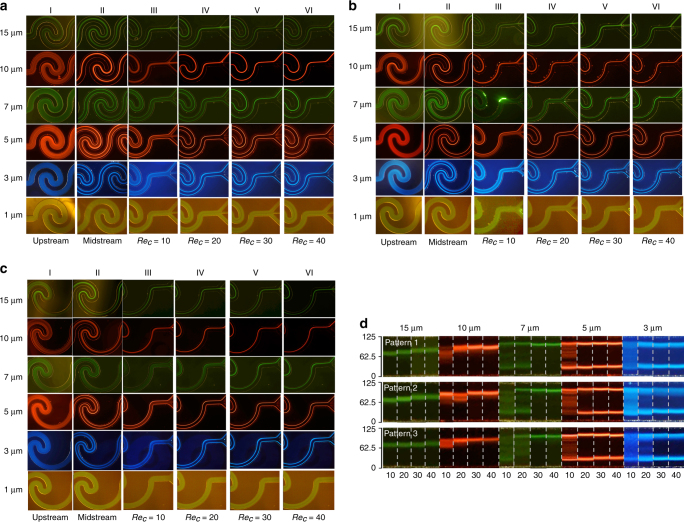
Fluorescence microscopic images of six differently sized particles (15, 10, 7, 5, 3, and 1 μm) undergoing inertial focusing in the three channel designs (a: Pattern 1; b: Pattern 2; c: Pattern 3). Columns I and II show the particle trajectory in the upstream and midstream, respectively. Columns III-VI show the particle trajectory in the downstream at Re_c_ = 10 (flow rate: 49.41 μl/min), 20 (flow rate: 98.83 μl/min), 30 (flow rate: 148.25 μl/min) and 40 (flow rate: 197.60 μl/min), respectively. **d** Comparison of the focusing position of the five particles in the downstream among the three channel designs. The abscissa and ordinate represent the channel Reynolds number Re_c_ and the channel width, respectively

We next investigated the inertial focusing behavior of differently sized microspheres (15, 10, 7, 5, 3 and 1 μm) in the three different channel designs. These microspheres are fluorescent, which allowed us to clearly visualize the particle trajectories even at very high flow rates. Figures 3a–c shows the fluorescent streak images of differently sized particles at varying flow rates in the three channel designs. Four different flow rates, 49.41, 98.83, 148.25 and 197.60 μl/min, were selected to study the inertial focusing behavior, which corresponds to channel Reynolds numbers Re_c_ = 10, 20, 30 and 40, respectively. We first focused on the inertial focusing behavior in channel pattern 1 (Fig. 3a). In the upstream of the channel (the first wavy channel unit, as shown in column I of Fig. 3a), the six differently sized fluorescent particles exhibited very similar behavior, fully occupying the entire channel cross-section without an obvious inertial focusing effect. In the midstream (column II of Fig. 3a), where the inertial focusing has not reached the steady state (Re_c_ = 40), the 15 and 10 μm particles revealed a tendency to focus along the centerline of the channel, whereas the 7, 5, and 3 μm particles tended to form two streaks near the two sidewalls of the channel. The smallest 1 μm particles showed no tendency to undergo inertial focusing. In the downstream (the last wavy channel unit, as shown in columns III to VI of Fig. 3a), in addition to the trifurcate outlets for the collection of the differently sized particles, these particles have reached their steady-state inertial focusing at different Re_c_. The 15 μm microspheres were focused to form a single streak along the centerline region of the channel even at Re_c_ = 10. As the inertial lift force near the central region scales with *U*^2^ and *a*^4^, achieving inertial focusing was easier with the 15 μm microspheres than with other particle sizes (*a*/*H* = 0.354>0.07). Another important parameter for evaluating the role of inertial lift force and Dean drag force is *R*_f_ = 0.354. In the following experiments, all the *R*_f_ values for the different particles were calculated using the radius of curvature along the outer wall of the inner semicircles (*R* = 175 μm). *R*_f_ = 0.354>0.08 implies that the inertial lift force dominates over the Dean drag force; as a result, the equilibrium focusing position of the 15 μm particles remained nearly along the centerline, similar to the inertial focusing along a straight channel with AR =  $h/w$ = 0.32. At Re_c_ = 10 and 20, the 15 μm microspheres eventually flowed into the middle outlet as expected. However, at Re_c_ = 30 and 40, the focused 15 μm microspheres flowed into the upper outlet, indicating that their equilibrium focusing position was slightly shifted toward the upper outlet. We speculate that the Dean drag force increased faster than the inertial lift force for the 15 μm microspheres as the flow rate increased. Although the Dean drag force was weaker than the inertial lift force, it would mildly shift the particle equilibrium focusing position away from the centerline.

The 10 μm microspheres (*a*/*H* = 0.165 > 0.07, *R*_f_ = 0.157>0.08) also focused into a single streak, with the inertial lift force dominating the focusing behavior. Additionally, the relatively weaker Dean drag force also shifted their equilibrium focusing position upward, and thus, the 10 μm microspheres flowed into the upper outlet. The inertial focusing behavior of the 7 μm microspheres, however, varied with the flow rates. *a*/*H* = 0.115 > 0.07 implies that they could be effectively focused. *R*_f_ = 0.077 is very close to the empirical value 0.08, indicating that the inertial lift force became comparable to the Dean drag force for the 7 μm microspheres. At Re_c_ = 10 and 20, the 7 μm microspheres were focused into two streaks near the sidewalls, where the Dean drag force slightly dominated the inertial lift force. Since the Dean secondary flow periodically reversed along the repeated wavy channel units, the Dean drag force tended to drag particles toward the two sidewalls. The balance between the Dean drag force and the inertial lift force, particularly the wall-induced lift force, led to equilibrium positions near the sidewalls. However, at Re_c_ = 30 and 40, the 7 μm microspheres were focused into a single streak shifted away from the centerline, revealing that the inertial lift force slightly dominated the Dean drag force. We speculate that 7 μm is at or very close to the threshold size at which the inertial lift force and Dean drag force became equally important. As the two forces varied with the flow rates in slightly different degree, the focusing behavior of the 7 μm microspheres could be readily switched between single streak focusing (shifted from the centerline) and double streak focusing (near the sidewalls). The two important parameters for the 5 μm microspheres are *a*/*H* = 0.083 > 0.07 and *R*_f_ =  0.039 < 0.08. Therefore, the 5 μm microspheres were predominantly affected by the Dean drag force and thus formed two streaks near the two sidewalls. For the 3 μm microspheres, although *a*/*H* = 0.049 < 0.07 (*R*_f_ = 0.014 < 0.08), they were still effectively focused into two streaks near the sidewalls. Note that this empirical parameter *a*/*H* = 0.07 for effective inertial focusing was obtained from a different channel design; thus, this threshold value may slightly deviate from 0.07 for different channel designs. For the 1 μm microspheres, the two important parameters are *a*/*H* = 0.016 < 0.07 and *R*_f_ = 0.002 < 0.08; therefore, they could evidently not achieve clear inertial focusing.

Figures 3b,c shows the inertial focusing behavior of the six differently sized particles in channel patterns 2 and 3, respectively. In general, the inertial particle focusing in the three channel designs followed quite similar tendencies, but the introduced geometric asymmetry in the lower outer semicircle still produced slight differences among the three designs. Figure 3d shows a clearer comparison of the focusing position and the width of the focusing streak for different particles in the three channels. For the 15 μm microspheres, when Re_c_ increased from 10 to 40, the focusing streak shifted from 64–80.5 to 73.37–89.87 μm in pattern 1, from 63–79.5 to 69.25–85.75 μm in pattern 2 and from 63–78 to 64.5–79.5 μm in pattern 3. As pattern 3 has the largest radius of curvature for the lower outer semicircle, the Dean secondary flow produced was slightly weakened, as also indicated by Table 1. Therefore, the 15 μm microspheres were preferably focused near the centerline in pattern 3 compared to the other two patterns. For the 10 μm particles, they were not fully focused at Re_c_ = 10. When Re_c_ increased from 10 to 40, the focusing streak shifted from 73.3–99.5 to 83.5–96.8 μm in pattern 1, from 63.5–86.5 to 83.2–96.5 μm in pattern 2 and from 81.5–97.8 to 87.5–97.5 μm in pattern 3. Therefore, pattern 3 was the best design to separate 15 μm particles from 10 μm particles, as the edge-to-edge distance between the focused streaks of the two particles was the largest (at least 8 μm). Figure 3c also shows that pattern 3 was the only design in which the 15 and 10 μm particles flowed into the middle outlet and the upper outlet, respectively, at all four Re_c_ values. For the 7 μm particles, they switched from double streak focusing (Re_c_ = 10 and 20) to single streak focusing (Re_c_ = 30 and 40) in pattern 1, as discussed previously. As the Dean secondary flow was weakened in pattern 2 and 3, the 7 μm particles were not very effectively focused at Re_c_ = 10 and 20. However, they exhibited single streak focusing at Re_c_ = 30 and 40 in patterns 2 and 3. The focusing streak of the 7 μm particles in the three patterns at Re_c_ = 30 and 40 were nearly identical, occupying 99.8–107.8 μm along the cross-section. The other three particles (5, 3, and 1 μm) behaved very similarly in the three channel patterns. The 5 μm particles displayed double streak focusing when Re_c_ ≥ 20, occupying 101.8–108.8 μm (upper streak) and 21.2–29.3 μm (lower streak) of the cross-section. Similarly, the 3 μm particles exhibited double streak focusing when Re_c_ ≥ 20, occupying 94.8–108.8 μm (upper streak) and 22–30.8 μm (lower streak). The 1 μm particles could not be effectively focused in any of the three channel patterns.

### Size-based inertial particle sorting

**Fig. 4:**
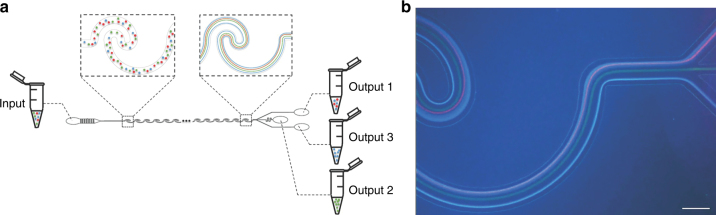
Separation of three differently sized particles using the pattern 3 sorting device. **a** Schematic experimental setup of the separation of three differently sized microparticles (3, 10 and 15 μm) in a single mixed input. **b** Fluorescent streaks of the three particles at the trifurcated outlets. The 10 μm (red fluorescence) and 15 μm particles (green fluorescence) were collected in output 1 and 2, respectively. The 3 μm particles (blue fluorescence) were collected in both output 1 and output 3. The flow rate was 197.60 μl/min (Re_c_ = 40). Scale bar is 125 μm

With knowledge of the inertial focusing behavior of individual particles with varying sizes in the three different patterns, we finally chose channel pattern 3 to demonstrate the sorting of a mixture of particles with multiple sizes. To achieve high throughput, a flow rate of 197.60 μl/min (Re_c_ = 40) was chosen for all the subsequent sorting experiments. Figure 4a shows the schematic experimental setup of the sorting of a particle mixture with 15 μm (green), 10 μm (red) and 3 μm (blue) fluorescent microparticles. The input particle mixture was sorted into three subpopulations collected by output 1, 2 and 3. Figure 4b shows the differential focusing of the three particles at the trifurcated outlets after flowing through a series of wavy channel units. These fluorescent streaks explicitly indicated that the 15 μm (green) particles were primarily focused along the centerline of the main channel and collected in output 2. The 10 μm (red) particles were focused into a tight streak near the upper edge of the main channel and thus were collected in output 1. In a previous study of the inertial focusing of individual particles, the edge-to-edge distance between the focusing streaks of 15 and 10 μm particles was approximately 8–10 μm. In this particle separation experiment, the distance between the focusing streaks of the 15 and 10 μm particles was increased to ~30 μm, which greatly favored the particle sorting. The enlarged separation distance between the 15 and 10 μm particles is likely attributable to the particle-particle interaction force at relatively high concentrations. When the larger 15 μm particles quickly occupied the middle region of the channel, these larger particles tended to repel smaller particles away from them. The smallest 3 μm (blue) particles were focused into two tight streaks close to the upper and lower edges of the main channel and accordingly collected in both output 1 and output 3. A video showing the particle separation process at the trifurcated outlets can be found as a Supplementary File (Supplementary Video S1).

### Size-based inertial cell sorting

**Fig. 5:**
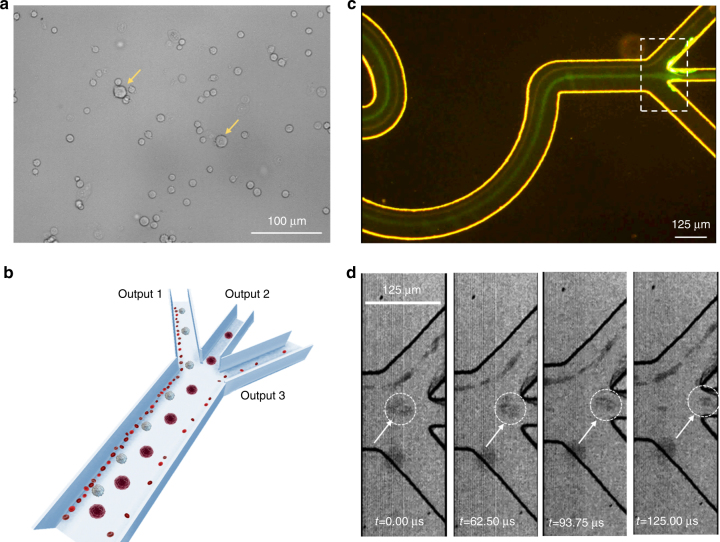
Separation of cancer cells from whole blood in the pattern 3 sorting device. **a** Microscopic image of individual cells in the mixture. The two yellow arrows indicate two individual MCF-7 cells that are much larger than the surrounding blood cells. Scale bar is 100 μm. **b** Schematic diagram showing the separation of cancer cells from diluted (100×) whole blood, in which the expected focusing positions of cancer cells, WBCs and RBCs are presented. **c** Fluorescence image of the focused cancer cells at the trifurcated outlets. Cancer cells are focused along the green line (exiting from output 2), and RBCs are focused along the dim red line (exiting from output 1). Scale bar is 125 μm. **d** The separation process of an individual MCF-7 cell from other blood cells captured by a high-speed camera in bright-field mode (the field of view is the dashed box shown in **c**). The arrows indicate the position of the MCF-7 cell at the corresponding times. Scale bar is 125 μm

We then used the pattern 3 inertial sorting device to separate breast cancer cells spiked in diluted whole blood samples in order to prove its potential clinical application in rare cell sorting. The whole blood sample was diluted 100 times using cell-free PBS buffer with a final concentration of 50 million cells per ml. The mixed cell samples, which contained ~5% fluorescently stained breast cancer cells (MCF-7, diameters of approximately 19–24 μm), were evaluated based on the fluorescence signal in flow cytometric analyses. The rest of the cell populations in the cell mixture were mainly red blood cells (RBCs, diameters of approximately 6–8 μm), platelets (diameters of ~3  μm) and white blood cells (WBCs, diameters of approximately 10–15 μm). Figure 5a shows a microscopic image of the mixed cell sample, in which individual MCF-7 cells appear much larger than the other blood cells. A flow rate of 197.60 μl/min (Re_c_ = 40) was used in order to achieve high throughput. According to the study of the inertial focusing of individual particles in Fig. 3, we expected that the MCF-7 cells would be collected in output 2, that a majority of WBCs and RBCs would be collected in output 1, and that platelets would be collected in both output 1 and 3, as shown in Fig. 5b.

The expected collection of different cell populations at the trifurcated outlets was verified through the sorting experiments presented in Fig. 5c,d. Figure 5c shows two major focusing streaks at the trifurcated outlets. Because of the relatively weak fluorescence of living cancer cells at a high-speed flow rate, only a dim green streak could be visualized to represent their focusing position. The high density of RBCs (in addition to the relatively lower amount of WBCs and platelets) even formed a focused dim red line without fluorescent labeling (the focusing streak of platelets near the lower edge of the channel could not be observed due to its small size and low density). Obviously, during the complete inertial focusing after flowing through a series of wavy channel units, breast cancer cells were concentrated along the centerline of the main channel and exhibited a large separation distance from RBCs, WBCs and platelets. A video of this cell separation process can be found as a supplementary file (Supplementary Video S2). Figure 5d even shows the separation process of an individual MCF-7 cell from other blood cells captured by a high-speed camera, in which a majority of blood cells flowed into output 1 and the larger MCF-7 cell (indicated by a white arrow) flowed into output 2 (Supplementary Video S3).

**Fig. 6:**
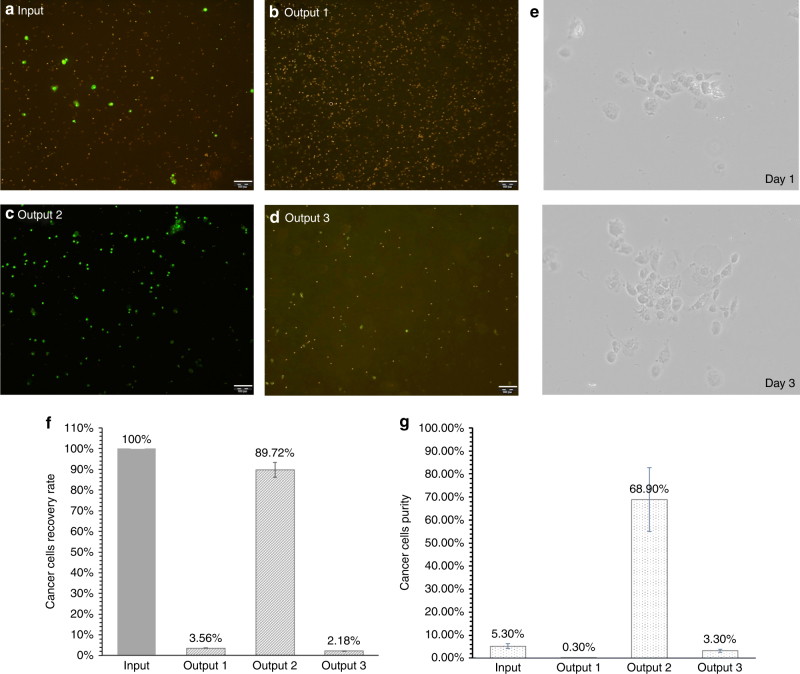
Microscopic images and flow cytometric results of pre-mixture and sorted samples. **a**–**d** Fluorescence image of diluted whole blood mixed with cancer cells from input to output through the inertial sorting device (Pattern 3). MCF-7 cells are indicated by green fluorescence. Scale bar is 100 μm. **e** MCF-7 cells collected from output 2 were cultured and were able to proliferate. **f** Recovery rate of MCF-7 cells through the inertial sorting device at the three outputs, which was obtained from flow cytometric analyses. **g** Purity of MCF-7 cells obtained from the inertial sorting device at the three outputs and inlet. Experiments were repeated five times

The original cell mixture and sorted samples collected from the three outputs were analyzed using a flow cytometer by counting at least 10,000 cells. Figures 6a–d shows microscopic images of the original cell mixture and the three samples collected from the outputs. The input sample contained fluorescently stained MCF-7 cells at a preset ratio of 5.3% with respect to whole blood cells. After the inertial sorting, almost all the MCF-7 cells were collected in output 2, as indicated by the concentrated green fluorescent spots in Fig. 6c. Output 1 collected a majority of the unlabeled blood cells (Fig. 6b), and only a small portion of blood cells were collected in output 3 (Fig. 6d). To quantitatively evaluate the sorting performance, we define the recovery rate of MCF-7 cells (Eq. 10) and purity of MCF-7 cells (Eq. 11) in each output,

10 $$Recovery\,rate = \frac{{Number\,of\,cancer\,cells\,in\,each\,output}}{{Number\,of\,cancer\,cells\,in\,input}}$$

11 $$Purity = \frac{{Number\,of\,cancer\,cells\,in\,each\,output/input}}{{Total\,number\,of\,cells\,in\,each\,output/input}}$$

After a single sorting process, 89.72% MCF-7 cells could be recovered from the original input sample, as shown in Fig. 6f. Due to the inevitable cell-cell interactions at high cell concentrations, a small portion of MCF-7 cells were also collected from output 1 and 3, as indicated by the recovery rates of 3.56% and 2.18% in outputs 1 and 3, respectively. Figure 6g shows the purity of MCF-7 cells in the three collected output samples after a single sorting process. The average purity of output 2 (isolated MCF-7 cells) is 68.9%, representing a 13 times purity enrichment compared to the original purity of 5.3%. We also studied the viability of MCF-7 cells collected from output 2. Figure 6e shows that the sorted MCF-7 cells were able to proliferate, indicating excellent cell viability after the inertial sorting process.

## Conclusion

In summary, we presented a new inertial focusing and sorting device with a series of reverse wavy channel structures that generate periodically reversed Dean secondary flow perpendicular to the main flow direction. The balance between two inertial effects, the inertial lift force and Dean secondary flow, produces size-dependent particle focusing across the channel. We experimentally studied the inertial focusing behavior of six particle sizes (15, 10, 7, 5, 3, and 1 μm) in three channel designs with different degrees of geometric asymmetry. It was found that when the inertial lift force dominated the Dean drag force for 15 and 10 μm particles, they exhibit single streak focusing. However, the different degree of force dominance for the 15 and 10 μm particles still resulted in distinct particle focusing positions. As the particle size shrunk, the two forces became comparable for 7 μm particles, which could switch from single streak focusing to double streak focusing at varying flow rates. When the Dean drag force dominated the inertial lift force for 5 and 3 μm particles, they were focused into two tight streaks near the two sidewalls. To achieve effective inertial focusing of even smaller particles (~1 μm), feasible methods include shrinking the dimensions of the channel cross-section and introducing viscoelastic force^55–57^ or other kinds of additional forces to facilitate particle focusing. Using the channel pattern 3 device, we demonstrated the separation of 15 μm particles from 10 and 3 μm particles. As the minimum particle size for effective inertial focusing is between 1 and 3 μm in channel pattern 3, we also demonstrated the separation of MCF-7 cancer cells from diluted whole blood samples without the use of sheath flows. We have found that a single sorting process was able to achieve an 89.72% recovery rate of MCF-7 cells from the original mixture and that the purity of MCF-7 cells was significantly increased from 5.3% to 68.9%. Sorted MCF-7 cells showed excellent viability and were able to proliferate. The linear array of these repeated wavy channel units enables easy horizontal (2D) and vertical (3D) parallelization of multiple channels, which provides great potential for high-throughput cell sorting in practical biomedical applications.

## Electronic supplementary material

S1_video1

S1_video2

S1_video3
